# Adolescent Brain Maturation and Cortical Folding: Evidence for Reductions in Gyrification

**DOI:** 10.1371/journal.pone.0084914

**Published:** 2014-01-15

**Authors:** Daniel Klein, Anna Rotarska-Jagiela, Erhan Genc, Sharmili Sritharan, Harald Mohr, Frederic Roux, Cheol E. Han, Marcus Kaiser, Wolf Singer, Peter J. Uhlhaas

**Affiliations:** 1 Department of Neurophysiology, Max-Planck Institute for Brain Research, Frankfurt am Main, Germany; 2 Ernst Strüngmann Institute (ESI) for Neuroscience in Cooperation with Max Planck Society, Frankfurt am Main, Germany; 3 Department of Neurocognitive Psychology, Institute of Psychology, Johann Wolfgang Goethe University, Frankfurt am Main, Germany; 4 Department of Psychiatry, Psychosomatic Medicine and Psychotherapy, Johann Wolfgang Goethe University, Frankfurt am Main, Germany; 5 Department of Brain and Cognitive Sciences, Seoul National University, Seoul, Republic of Korea; 6 School of Computing Science and Institute of Neuroscience, Newcastle University, Newcastle, United Kingdom; 7 Frankfurt Institute for Advanced Studies, Johann Wolfgang Goethe University, Frankfurt am Main, Germany; 8 Institute of Neuroscience and Psychology, University of Glasgow, Glasgow, United Kingdom; University of Montreal, Canada

## Abstract

Evidence from anatomical and functional imaging studies have highlighted major modifications of cortical circuits during adolescence. These include reductions of gray matter (GM), increases in the myelination of cortico-cortical connections and changes in the architecture of large-scale cortical networks. It is currently unclear, however, how the ongoing developmental processes impact upon the folding of the cerebral cortex and how changes in gyrification relate to maturation of GM/WM-volume, thickness and surface area. In the current study, we acquired high-resolution (3 Tesla) magnetic resonance imaging (MRI) data from 79 healthy subjects (34 males and 45 females) between the ages of 12 and 23 years and performed whole brain analysis of cortical folding patterns with the gyrification index (GI). In addition to GI-values, we obtained estimates of cortical thickness, surface area, GM and white matter (WM) volume which permitted correlations with changes in gyrification. Our data show pronounced and widespread reductions in GI-values during adolescence in several cortical regions which include precentral, temporal and frontal areas. Decreases in gyrification overlap only partially with changes in the thickness, volume and surface of GM and were characterized overall by a linear developmental trajectory. Our data suggest that the observed reductions in GI-values represent an additional, important modification of the cerebral cortex during late brain maturation which may be related to cognitive development.

## Introduction

A large body of work during last two decades has highlighted the importance of adolescence for the continued maturation of cortical circuits [1]–[3]. Starting with the observation of Huttenlocher [4] of marked decreases in the number of synaptic contacts, magnetic resonance imaging (MRI) studies have disclosed pronounced reductions in the volume and thickness of gray matter (GM) [5], [6]. In contrast, the amount of white matter (WM) has been shown to increase as a result of improved myelination of cortico-cortical connections [7]–[10]. More recent research has indicated that modifications in GM/WM extend into the third decade of life [11], [12] and involve changes in the large-scale organization of anatomical and functional networks [13]. These findings provided novel insights into the importance of adolescence as a critical period of human brain development which may also hold important clues for the emergence of psychiatric disorders, such as schizophrenia, which typically manifest during the transition from adolescence to adulthood [14], [15].

While modifications in the volume of GM/WM have been extensively characterized, relatively little evidence exists on maturational changes in the folding of the cortical surface. The cerebral cortex in humans has as one of its distinguishing characteristics a highly convoluted folding pattern that leads to a significantly increased cortical surface. For example, the surface area of the human cortex is per average ten times larger than that of the macaques monkey but only twice as thick [16]. The increased cortical surface in humans may be related to the emergence of higher cognitive functions because of the large number of neurons and cortico-cortical connections that can be accommodated.

There is evidence that the cortical folding pattern is subject to developmental changes. After 5 months in utero, cortical folds appear and continue to develop at least into the first postpartum year [17]. During early childhood, the degree of gyrification further increases and has so far been assumed to stabilize thereafter. Post-mortem analyses by Armstrong et al. [18], however, observed a significant overshot in cortical folding until the first year followed by a reduction until adulthood.

This finding is supported by recent MRI-studies which have investigated GI-values during brain maturation. Raznahan et al. [19] demonstrated a global decrease in gyrification during adolescence. More recently, Mutlu et al. [20] showed that GI-values declined between 6–29 years of age in frontal and parietal cortices which is consistent with data from Su and colleagues [21] who applied a novel approach of gyrification measurement towards a small sample of children and adolescents. Finally, data by Hogstrom et al. [22] suggest that modfications in gyrification continue until old age.

In the present study, we sought to comprehensively characterize the development of gyrification during adolescence through investigating whole-brain GI-values in MRI-data. In addition, we obtained GM-parameters (cortical thickness, volume and surface area) as well as WM-volume estimates to determine the relationship between age-dependent changes in gyrification and GM/WM parameters. Our results show widespread reduction in GI-values which occur in overlapping but also distinct areas of GM-change, such as in precentral, temporal and frontal regions, which highlight the ongoing anatomical modification of the cerebral cortex during adolescence.

## Materials and Methods

### Participants

85 right-handed participants (36 males and 49 females) between the ages of 12 and 23 years were recruited from local high-schools and the Goethe University Frankfurt and were screened for the presence of psychiatric disorders, neurological illness and substance abuse. Written informed consent was obtained from all participants. For participants younger than 18 years, written consent was given by their parents. The Hamburger-Wechsler intelligence testing battery (HAWI-E/K) [23], [24] was performed. Six participants were excluded cause of lacking or incomplete MRI-data. The study was approved by the ethics board of the Goethe-University Frankfurt.

### MR Data Acquisition

Structural magnetic resonance images were obtained with a 3-Tesla Siemens Trio scanner (Siemens, Erlangen, Germany), using a CP head coil for RF transmission and signal reception. We used a T1-weighted three-dimensional (3D) Magnetization Prepared Rapid Acquisition Gradient Echo (MPRAGE) sequence with the following parameters: time repetition (TR): 2250 ms., time echo (TE): 2.6 ms., field of view (FOV): 256×256 mm^3^, slices: 176 and a voxel size of 1×1×1.1 mm^3^.

### Surface reconstruction

MRI-data were processed with the surface and volume pipeline of the FreeSurfer-software version 5.1.0 (http://surfer.nmr.mgh.harvard.edu) [25], [26] and estimates of cortical thickness, GM- and WM- volume, cortical surface area, the 3-D local gyrification Index (lGI) and estimated intracranial volume (eTIV) were obtained. The standard FreeSurfer pipeline was followed and automatically reconstructed surfaces were inspected for accuracy and if necessary, manual interventions using FreeSurfer correction tools were used.

Pre-processing included Talairach transformation, motion correction, intensity normalization, non brain tissue removing, segmentation and tessellation of the gray and white matter boundary, automatically topology correction and surface deformation and is described in more detail elsewhere [25], [27]–[29]. In addition, a spherical atlas registration, inflation and a gyral/sulcal based parcellation of the cortical surface was performed for inter-individual analyses which yielded 33 cortical areas per hemisphere [30].

### Cortical thickness, Cortical Surface Area and GM-volume

Cortical thickness was measured as the distance between the WM- boundary and the GM-matter surface at each point (vertex) on the tesselated surface [27]. Cortical surface area maps were generated through area estimations of each triangle in a standardized surface tessellation [31]. Area estimations were mapped back to the individual cortical space by means of a spherical atlas registration [32]. This yielded vertex-by-vertex estimates of the relative areal expansion or compression [33]. Estimates of GM-volume were derived from cortical thickness measures and the area around the corresponding vertex on the cortical surface [34].

### 3-D local gyrification index (lGI)

A 3-D lGI was computed [35] which has been employed in previous MR-studies [36], [37]. In brief, the lGI involves a 3-D reconstruction of the cortical surface where the degree of gyrification is defined as the amount of cortex surface buried within the sulcal folds compared with the amount of visible cortex in circular regions of interest [38]. In the first step, a triangulated outer surface which tightly wraps the pial surface was created through a morphological closing procedure. After converting the pial mesh into a binary volume, we used a diameter of 15 mm to close the main sulci for for generating the sphere [35]. For creating the circular region of interest (ROI), we choose a radius of 25 mm to include more than one sulcus to obtain an optimal resolution [38]. The initial lGI-values of a vertex were defined as the ratio between the surface of the outer ROI and the surface on the pial surface. For statistical comparisons, the outer lGI-values were mapped back to the individual coordinate system which reduced interindividual sulcal misalignment [35].

### WM-volume

The regional WM-volume below parcellated cortical GM-regions were estimated. Each white matter voxel was labeled to the nearest cortical GM-voxel with a distance limit of 5 mm resulting in 33 WM-volumes of the corresponding 33 gyral labeled GM areas [39] which has been used in previous studies [9], [40].

### Estimated intracranial volume (eTIV)

The estimated intracranial Volume (eTIV) in the FreeSurfer pipeline was derived from an atlas normalization procedure. Through the Atlas Scaling Factor (ASF), which represents a volume-scaling factor to match an individual to an atlas target, calculations of each eTIV were performed [41].

### Statistical Analysis

**Figure 1:**
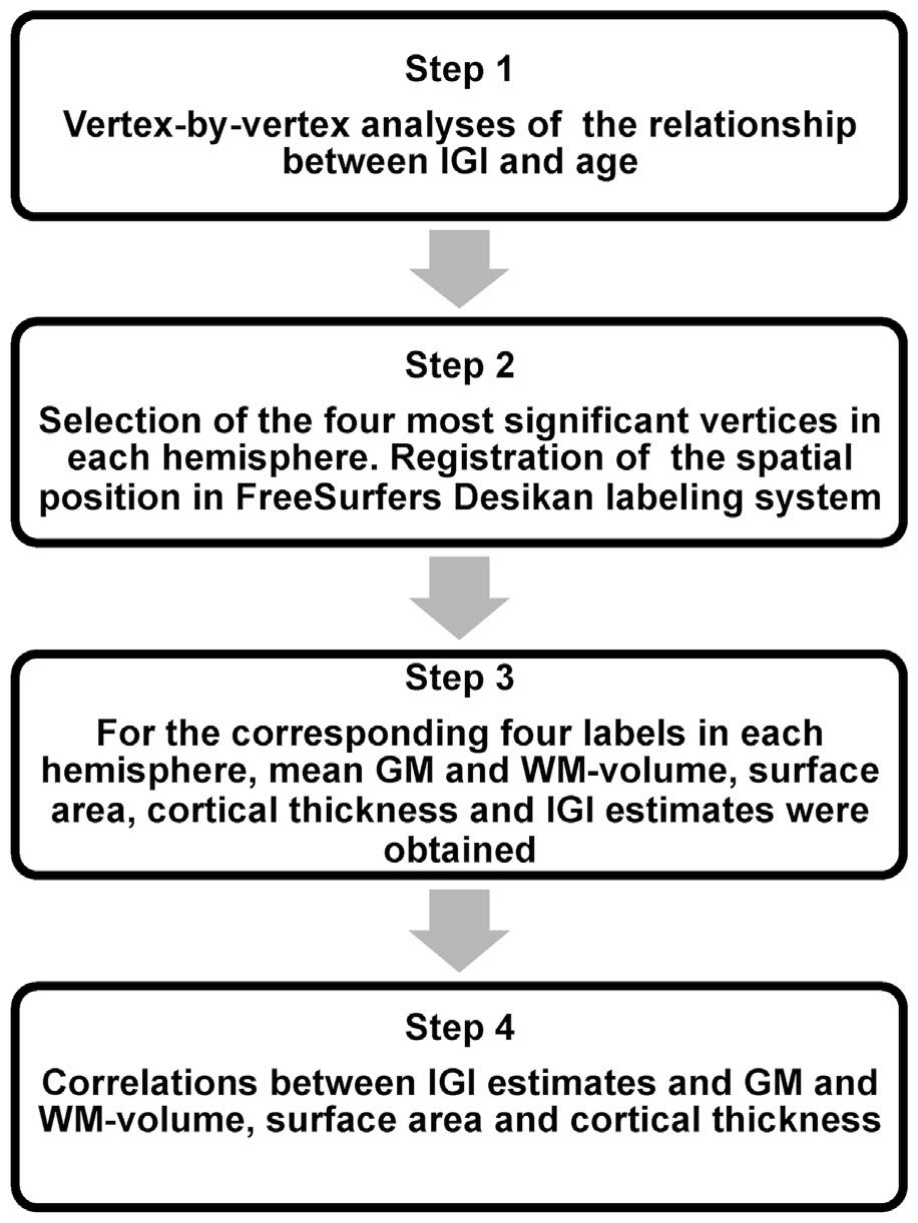
Analyses Steps for the lGI-Values and Correlations with Anatomical Parameters (GM/WM-volume, Cortical Surface Area and Cortical Thickness).

The analyses steps are summarized in Figure 1. Surfaces of the right and left hemispheres of all 79 participants were averaged and individual surfaces were resampled into the average spherical coordinate system. To increase the signal to noise ratio, we used 20 mm full-width at half maximum (FWHM) smoothing for the estimation of cortical thickness, GM- volume and cortical surface area and 5 mm FWHM for the lGI.

In the first step, we investigated whole-brain lGI-values, cortical thickness, cortical surface area and volume of GM in a vertex-by-vertex analysis. A General Linear Model (GLM) was employed to analyze the effect of age on the different anatomical parameters (lGI, cortical thickness, cortical surface area and GM-volume). All analyses were performed while controlling for the effects of gender and eTIV. We employed a false discovery rate approach (FDR) [42] to correct for multiple comparisons with a criterion for cortical thickness, surface area and GM-volume of q 0.05 and q 0.005 for lGI estimates. Different statistical thresholds were chosen because of the widespread, age-dependent changes in lGI-values compared to cortical thickness, cortical surface area and GM-volume. In addition we analyzed age^2^ and age^3^ effects for all anatomical parameters which were controlled for the influence of age, gender and eTIV.

To obtain estimates of area size, we selected vertices with the largest lGI-values and their corresponding Talairach coordinates and applied the automatic mri_surfcluster function in FreeSurfer (http://surfer.nmr.mgh.harvard.edu/fswiki/mri_surfcluster). In addition, Cohen's d [43] was obtained for brain areas with the largest age-dependent changes through the comparison between the mean values in the youngest (age: 12–14, n = 13) and oldest participant group (age: 21–23, n = 18). Effects sizes are reported in figure legends.

In a second step, we examined Pearson correlation coefficients between age-dependent lGI-effects and changes in cortical thickness, cortical surface area and GM/WM-volume. To include WM-volume data, parcellation based regional analyses were performed. Four vertices from the vertex-by-vertex analyses per hemisphere with pronounced age-lGI effects (statistical threshold p<10^−4^) were assigned to FreeSurfers gyral based areas [30] and for the corresponding labels mean cortical thickness, GM/WM-volume and cortical surface area were extracted.

## Results

### Vertex-by-vertex analyses of age-dependent changes in lGI

**Figure 2:**
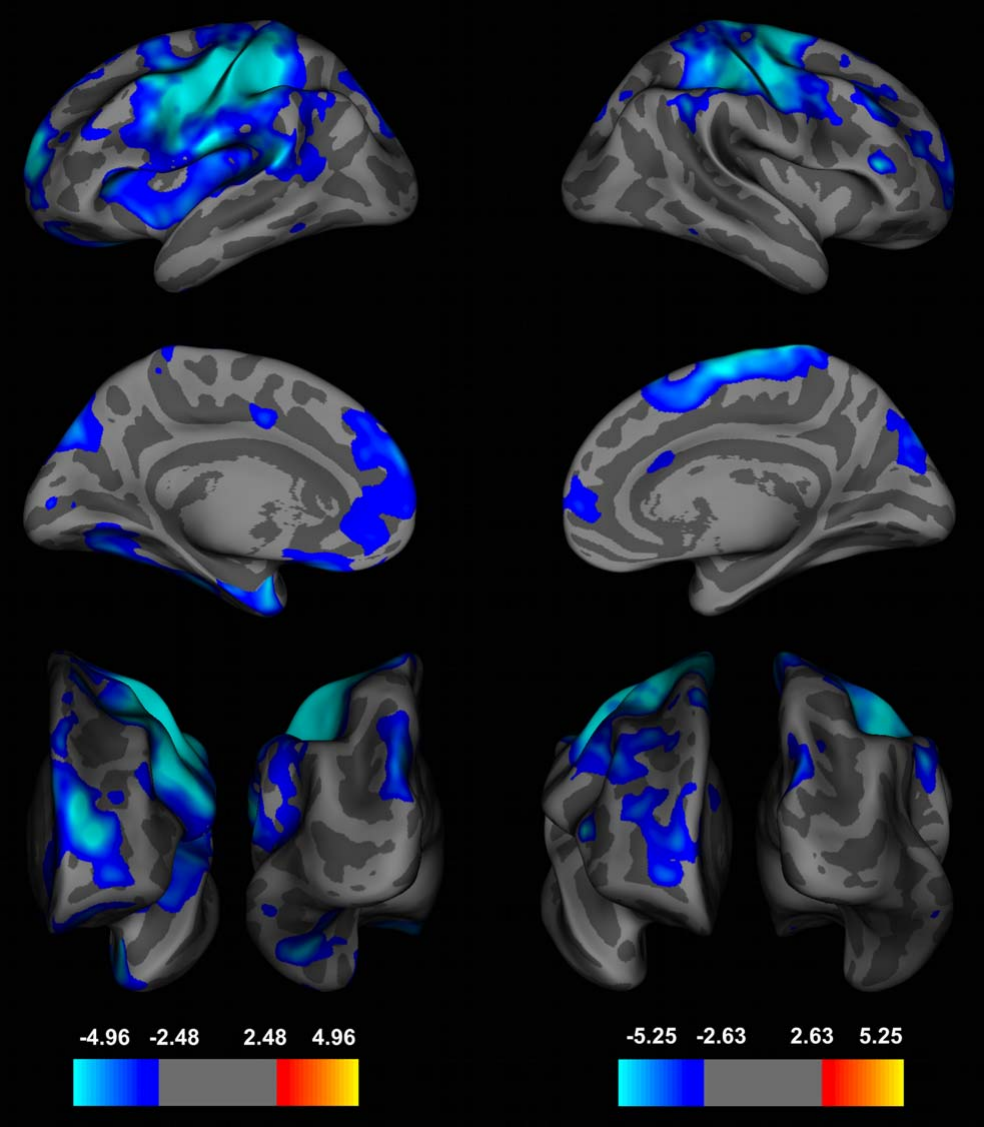
Whole-Brain Analyses of the Local Gyrification Index (lGI) during Adolescence. Effects of age on the local gyrification index (lGI) in a whole brain, vertex-by-vertex analyses projected onto an average template brain. Left panel: left hemisphere from lateral (top), medial (middle), frontal (left bottom) and occipital (right bottom) view. Right panel: right hemisphere from lateral (up), medial (middle), frontal (left bottom) and occipital (right bottom) view. Blue colors indicate a significant decrease of lGI-values with increasing age, whereas warmer colors are coded for an increase in lGI. Age-dependent effects were corrected for multiple comparisons with a false discovery rate (FDR) of q at 0.005 and a smoothing of 5 mm was used. lGI-values showed the largest age-dependent effects in precentral, frontal and parietal regions (left hemisphere (effect sizes, Cohen's d): precentral cortex: d = 1.61, superior-frontal cortex: 1.83, lateral-orbitofrontal cortex: d = 1.06, superior parietal cortex: d = 1.20; right hemisphere: precentral cortex: d = 1.57, rostral-middle frontal gyrus: d = 1.79, pars triangularis: d = 1.36, inferior parietal cortex: d = 1.58).

**Figure 3:**
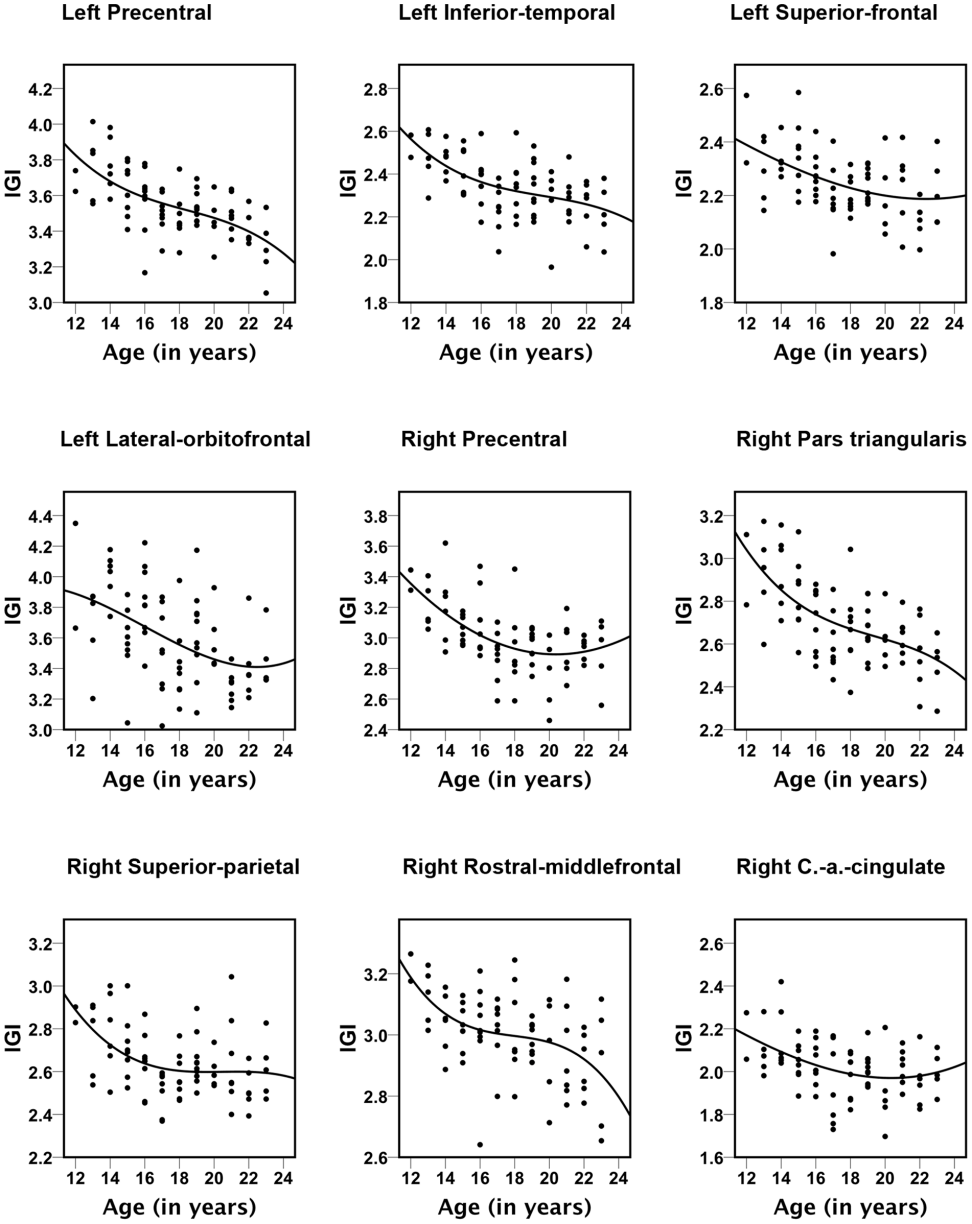
Scatter plots for the nine brain areas with significant correlations between age and lGI-values. All trajectories were best represented with cubic fits. The explained variance (R^2^) values are: left Precentral = 0.396, left Inferior-temporal = 0.218, left Superior-frontal = 0.298, left Lateral-orbitofrontal = 0.196; right Precental = 0.382, right Pars triangularis = 0.228, right Superior-parietal = 0.217, right Rostral-middlefrontal = 0.311, right Caudal-anterior (a.) Cingulate = 0.161.

lGI-values decreased with age in 12 clusters in the left and 10 clusters in the right hemisphere (FDR at 0.005) (Figure 2 and 3, Table 1). Brain areas with the largest lGI-reductions were localized to the left precentral (area size = 22211.63 mm^2^, p = 10^−8.42^, BA 6 and 7), left superior-frontal (area size = 3804.76 mm^2^, p = 10^−5.69^, BA 10), left inferior-temporal (area size = 2477.53 mm^2^, p = 10^−4.61^, BA 19, 20 and 37), left lateral-orbitofrontal (area size = 1834.36 mm^2^, p = 10^−4.45^, BA 47 and 11) and right precentral cortex (area size = 12152.39 mm^2^, p = 10^−7.47^, BA 6 and 7), right pars triangularis (area size = 271.76 mm^2^, p = 10^−4.57^, BA 10 and 46), right rostral-middlefrontal (area size = 1200.69 mm^2^, p = 10^−4.57^, BA 9) and superior parietal (area size = 1834.36 mm^2^, p = 10^−4.26^, BA 19 and 39). No significant effects of gender were found for changes in lGI-values at a FDR at 0.005 and age-related reductions in gyrification followed nonlinear (cubic) trajectories (Figure 3).

**Table 1:** Age-Related Decreases in Gyrification.

Hemisphere	Cluster	Max.	max. Vertex	Area size	Talairach coordinates			Number	Anatomical	BA
	number	p-value		(in mm^2^)	x	y	z	of vertices	region	
	1	10^−7.47^	13453	12152.39	20.7	−13.3	6.6	47118	Precentral	6/7
	2	10^−5.29^	65514	271.76	48.3	30.8	12	5248	Pars triangularis	46/10
	3	10^−4.57^	26871	1200.69	37.7	28.9	36.9	1515	Rostral-middle frontal	9
	4	10^−4.35^	93087	1896.57	21.6	62.8	9	3467	Rostral-middle frontal	10
Right	5	10^−4.26^	98674	1834.36	10.3	−86.1	37.9	2745	Superior-parietal	19/39
	6	10^−3.36^	118818	959.69	5.3	21.3	25.2	2537	Caudal-anterior cingulate	24
	7	10^−3.30^	76024	91.74	22.4	17.2	55.8	432	Superior-frontal	6
	8	10^−3.27^	70292	41.41	60.6	−47.5	−20	76	Inferior-temporal	37
	9	10^−3.09^	157640	23.99	20.1	−68.1	39.2	102	Superior-parietal	7
	10	10^−3.06^	53884	47.96	13.1	26.1	−14.8	109	Lateral-orbito frontal	25
	1	10^−8.42^	58054	22211.63	−33.3	−22.5	46.4	47118	Precentral	3/6
	2	10^−5.69^	138184	3804.76	−18.4	59.1	18.4	5248	Superior-frontal	10
	3	10^−4.61^	15700	2477.53	−91.8	−24.4	−21.7	3467	Inferior-temporal	20/19/37
	4	10^−4.45^	6055	1834.36	−14.2	19.3	−24.8	2745	Lateral-orbito frontal	47/11
	5	10^−4.20^	147348	1575.41	−18.1	−76.3	34.9	2537	Superior-parietal	7
Left	6	10^−3.61^	56475	145.11	−10.7	0	40.9	432	Posterior-cingulate	24
	7	10^−3.46^	158239	67.02	−7.3	−89.1	5.5	76	Pericalcarine	17
	8	10^−3.23^	53258	85	−57.6	53.5	−129.3	102	Inferior-temporal	37
	9	10^−3.21^	128857	83.29	−26	42.8	17.7	109	Rostral-middle frontal	10
	10	10^−3.13^	49868	310.34	−38.1	34.7	29.8	457	Rostral-middle frontal	9
	11	10^−3.11^	46342	47.91	−41.6	−5.7	−44.5	72	Inferior-temporal	20
	12	10^−2.99^	82816	63.25	−12.8	26.6	54.8	82	Superior-frontal	6

Note: Reported are cluster number, maximum p-value within a cluster, maximum vertex, area size of the cluster (in mm^2^), Talairach coordinates of the vertex, number of vertices, FreeSurfers anatomical region, Brodman area (BA). Note some clusters span more than one anatomical region or BA. Neuroanatomical classifications were done by Talairach Client v.2.4.2 (http://www.talairach.org/client.html) and FreeSurfers Desikan gyral based atlas (Desikan et al., 2006).

### Vertex-by-vertex analyses of age-dependent changes in Cortical Thickness, GM-Volume and Cortical Surface Area

**Figure 4:**
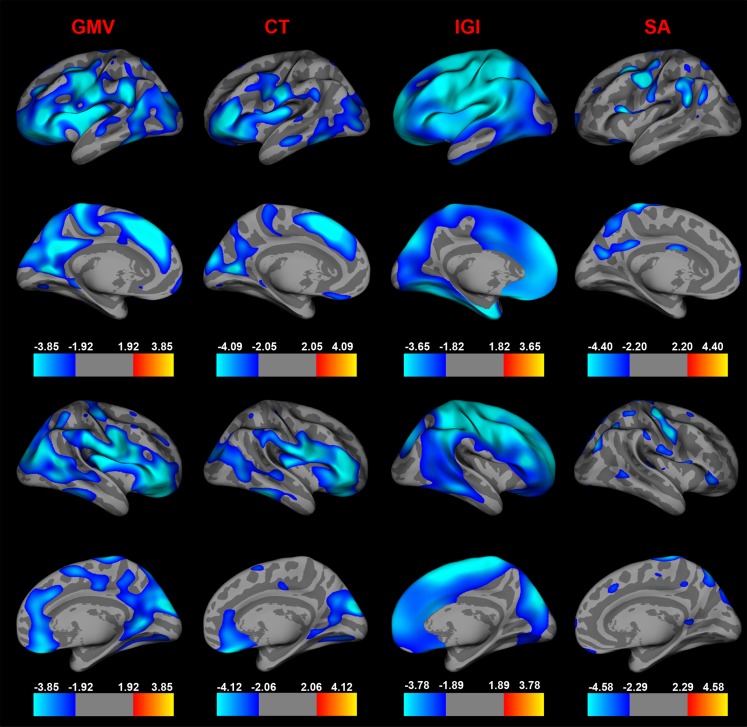
Comparison of Age-Related Changes between GM-Volume, Cortical Thickness, Cortical Surface Area and Gyrification. Age effects in a vertex-by-vertex analyses on GM-volume (GMV), cortical thickness (CT), lGI and cortical surface area (SA) presented on an average template brain. Left hemisphere from lateral view in the first row, from medial view in the second row. Right hemisphere is viewed from lateral view in the third row; medial view in the fourth row. The analyses for all parameters (lGI, cortical thickness, GM-volume and cortical surface area) were corrected for multiple comparisons with a false discovery rate (FDR) of q at 0.05 and to increase the signal to noise ratio, a 20 mm full-width at half maximum (FWHM) smoothing was employed. Blue colors indicate a significant decrease of lGI-, cortical thickness-, GM-volume- and cortical surface area- values with increasing age, whereas warmer colors are coded for age-related increases. Cortical thickness reductions showed the largest age-dependent effects in frontal, temporal and parietal regions (left hemisphere (effect sizes, Cohen's d): superior-frontal cortex: d = 2.27, rostral-middle frontal cortex: 1.84, pericalcarine: d = 1.29, middle-temporal gyrus: d = 1.01; right hemisphere: precentral cortex: d = 1.07, cuneus: d = 1.07, inferior temporal cortex: d = 1.15, superior frontal gyrus: d = 0.56) which overlapped with decreased GM-volume (left hemisphere (effect sizes, Cohen's d): superior-frontal: d = 0.78, superior-temporal: 1.46, lingual gyrus: d = 0.48, post-central gyrus: d = 0.81; right hemisphere: pars orbitalis: d = 0.59, inferior parietal: d = 1.39, paracentral gyrus: d = 0.82, superior frontal: d = 1.12). Cortical surface area decreased most prominently in parietal, precentral, supramarginal and caudal-middle frontal cortices (left hemisphere (effect sizes, Cohens'd): precentral cortex: d = 0.7, caudal-middle-frontal: d = 0.34, supramarginal: d = 0.45; right hemisphere: precentral cortex: d = 0.67, superior parietal: d = 0.69 and inferior parietal: d = 1.01).

Cortical thickness decreased most prominently in the superior-frontal (area size = 2608.63 mm^2^, p = 10^−7.13^, BA 6, 8 and 9) and rostral-middle-frontal (area size = 12859.08 mm^2^, p = 10^−6.08^, BA 11, 44, 45 and 46) cortices in the left hemisphere and in the precentral cluster in the right hemisphere (area size = 14735.38 mm^2^, p = 10^−6.16^, BA 6, 44 and 45) (Figure 4). The cortical thickness decrease can be described by a cubic trajectory (R^2^ = 0.191 for left rostral-middle-frontal, R^2^ = 0.126 for left superior-frontal and R^2^ = 0.134 for right precentral clusters). Moreover, we found age-dependent, bilateral decreases in GM-volume which were localized to the superior-frontal (area size = 45212.15 mm^2^, p = 10^−7.60^, BA 6, 8 and 9) lobe in the left hemisphere and to the pars orbitalis (area size = 19200.11 mm^2^, p = 10^−6.68^, BA 44, 45 and 47) and to the inferior-parietal (area size = 16614.72 mm^2^, p = 10^−5.03^ BA 19 and 39) lobe of the right hemisphere (Figure 4). GM-volume reductions followed cubic trajectories (R^2^ = 0.132 for left superior-frontal, R^2^ = 0.185 for right pars orbitalis and R^2^ = 0.204 for right inferior parietal clusters).

For surface area, we found a significant reduction in precentral (area size = 2296.99 mm^2^, p = 10^−9.64^, BA 4), caudal middle frontal (area size = 609.mm^2^, p = 10^−6.03^, BA 6) and supramarginal (area size = 1647.24 mm^2^, p = 10^−4.88^, BA 22) clusters in the left hemisphere. Surface area decreased in the right hemisphere most prominently in precentral (area size = 1371.37 mm^2^, p = 10^−6.34^, BA 4), inferior parietal (area size = 1248.36 mm^2^, p = 10^−5.99^, BA 7) and superior parietal (area size = 652.77 mm^2^, p = 10^−4.11^, BA 7) cortices (Figure 4). Reductions in surface area were best described by a cubic trajectory (R^2^ = 0.095 for left precentral, R^2^ = 0.026 left caudal-middle frontal, R^2^ = 0.024 left supramarginal, R^2^ = 0.116 right hemisphere, R^2^ = 0.156 right superior-parietal and R^2^ = 0.046 for right precentral clusters). No significant effects of gender were found for changes in cortical thickness, GM-volume and surface area at a FDR at 0.005

### Correlations between Gyrification, Cortical Thickness, Surface Area and GM/WM-Volume

**Figure 5:**
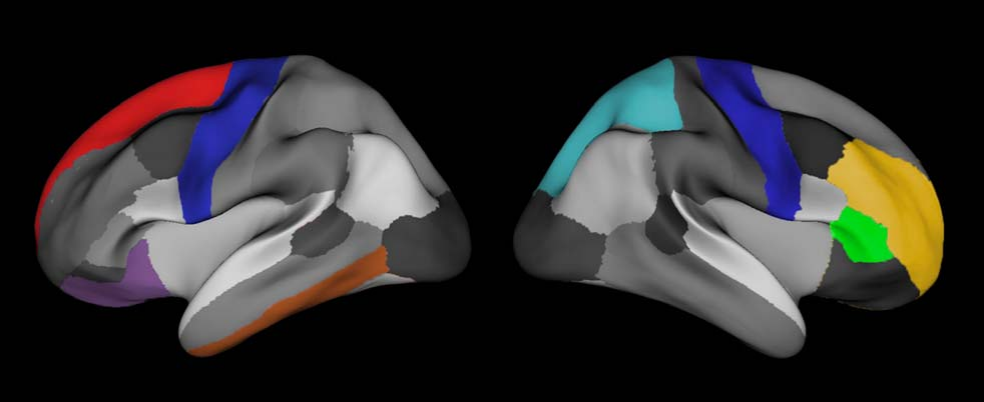
Based on the FreeSurfers Desikan labeling, eight regions of interest (ROI's) were selected to analyze the relationships between lGI, Cortical Thickness, GM-volume, Cortical Surface Area and WM-volume. ROI's in the left hemisphere (lateral view): 1) precentral gyrus (blue), 2) superior frontal gyrus (red), 3) lateral-orbitofrontal cortex (violet) and 4) inferior temporal cortex (brown). ROI's right hemisphere (lateral view): 1) precentral gyrus (blue), 2) rostral-middle-frontal gyrus (yellow), 3) pars triangularis gyrus (green) and 4) superior-parietal cortex (light blue). Note the relative good spatial orientation in comparison to brain areas, which lGI's are significant correlated with age from Figure 4.

To test for relationships between lGI-values and changes in GM/WM, 8 areas with the largest age-dependent changes in gyrification were selected and lGI-values were correlated with cortical thickness, cortical surface area and GM/WM-Volume (Figure 5, Table 2). We found large and positive correlations between cortical surface area and GM-volume with lGI-values. Such a relationship was not found for correlations between cortical thickness and lGI-estimates. Increased WM-volume also showed a significant albeit weaker relationship than GM-volume and surface area with enhanced gyrification in several frontal regions and in parietal cortex.

**Table 2:** Correlations between mean lGI-Values with Thickness, WM-, GM-Volume and Surface Area.

Region of Interest	Thickness	WM-Volume	GM-Volume	Surface area
**Left Hemisphere:**				
Precentral Gyrus	0.09	0.03	0.43 ***	0.42 ***
Superior-frontal Gyrus	−0.10	0.30 **	0.60 ***	0.64 ***
Inferior-temporal Gyrus	−0.20	0.15	0.44 ***	0.55 ***
Lateral-orbitofrontal cortex	−0.02	0.31 **	0.50 ***	0.55 ***
**Right Hemisphere:**				
Precentral Gyrus	0.04	0.16	0.36 **	0.37 **
Rostral-middle frontal gyrus	−0.11	0.39 ***	0.67 ***	0.74 ***
Pars triangularis Gyrus	0.17	0.25 *	0.36 ***	0.31 *
Superior-parietal Cortex	0.17	0.28 **	0.52 ***	0.55 ***

r. values with p<0.05.

p<0.01.

p<0.001.

### Non-Linear Relationships between Changes in Anatomical Parameters and Age: A Vertex-by-Vertex Analyses

#### lGI

We found 16 (left hemisphere) and 7 Clusters (hemisphere) where age^2^ and lGI were negatively correlated (Figure S1). The strongest age ^2^ effects on lGI were localized in left superior-frontal (area size = 2147.01 mm^2^, p = 10^−5.48^, BA 8, 9 and 10), left superior-parietal (area size = 5233.35 mm^2^, p = 10^−4.51^, BA 1, 2, 3, and 4) and left pericalcarine (area size = 243.34 mm^2^, p = 10^−3.80^, BA 17) clusters. For the right hemisphere, effects were observed in a precentral region (area size = 1165.59 mm^2^, p = 10^−4.81^, BA 1, 2, 3, 4, and 6), postcentral (area size = 465.07 mm^2^, p = 10^−3.53^, BA 1, 2 and 3) and in superiorfrontal cortices (area size = 330.55 mm^2^, p = 10^−3.48^, BA 8).

Cubic effects of age on lGI were found in 18 (left hemisphere) and 7 Clusters (right hemisphere). Regions with the strongest cubic effects were localized in a large superior-frontal (area size = 5598.96 mm^2^, p = 10^−6.54^, BA 8, 9, 10, 11, 45, 46 and 47), superior-parietal (area size = 11513.02 mm^2^, p = 10^−6.11^, BA 1, 2, 3, 4, 5, 6, 7, 8 and 9) and pericalcarine (area size = 292.35 mm^2^, p = 10^−3.73^, BA 17) cluster for the left hemisphere. In the right hemisphere, strongest cubic age and lGI relations were found in a precentral (area size = 5862.33 mm^2^, p = 10^−5.52^, BA 6, 4, 5, and 7), caudal-middlefrontal (area size = 503.66 mm^2^, p = 10^−3.56^, BA 8 and 9) and middle-temporal cluster (area size = 152.44 mm^2^, p = 10^−2.98^, BA 21).

#### GMW

Age^2^ effects on GMV were confined to the left hemisphere (Figure S2). Strongest effects were seen in extended parts of the pars opercularis (area size = 630.89 mm^2^, p = 10^−4.35^, BA 13, 44 and 45), paracentral (area size = 495.23 mm^2^, p = 10^−4.11^, BA 4, 6 and 31) and inferior-parietal (area size = 144.45 mm^2^, p = 10^−3.71^, BA 39 and 22) cortices.

Cubic age effects on GMV were located in 3 cortices in the left hemisphere. One cluster in the posterior parts of the gyrus cinguli (area size = 175.00 mm^2^, p = 10^−4.55^, BA 31), a part of the gyrus inferior frontalis-pars opercularis- (area size = 124.78 mm^2^, p = 10^−4.25^, BA 44) and the banks of superior temporal sulcus (area size = 7.12 mm^2^, p = 10^−3.61^, BA 39) were characterized by a significant age^3^ and lGI relationship (Figure S2).

CT/SA: No significant age^2^/age^3^ effects we found for CT and SA.

## Discussion

The results of our study highlight widespread changes in the gyrification-pattern of the cerebral cortex during adolescence. Previous post-mortem [18] and MRI-studies [19]–[21] indicated a decrease of lGI-values during later developmental periods but the extent of change, the brain regions involved and the relationship with concurrent anatomical process have remained unclear. Cortical areas which were characterized by the strongest reductions in lGI-values were precentral, temporal and frontal regions. These brain areas overlapped only partially with regions characterized by changes in GM and effect sizes were in the range and above for cortical thickness and GM-volume, suggesting that the observed modifications in gyrification represent an additional, important modification of the cerebral cortex during adolescence.

### Cortical Regions of IGl-Changes

The largest cortical region characterized by reductions in gyrification was a cluster in the precentral cortex which included BA 3, 6 and 7. In comparison, changes in the thickness and volume of GM were focused over frontal (BA 8 and 9) and temporal (BA 20 and 21) cortices, which is consistent with data from previous longitudinal studies [6] but overlapped only partially with decreased lGI-values.

Although the precentral cluster, which extended to pre-/post-central gyrus, supramarginal gyrus as well as to the superior parietal cortex, has been less consistently involved in adolescent brain maturation, there is evidence to suggest that these brain areas may be related to ongoing changes in cognition and behavior. A recent study by Ramsden et al. [44] demonstrated that fluctuations in intelligence during adolescence are closely related to GM-changes in left motor speech regions. Similarly, there is ongoing improvement in motor cortex as revealed through studies with transcranial magnetic stimulation (TMS) [45] and EEG [46]. Finally, BA 7 is critical for the developmental of cortical networks underlying higher cognitive functions during adolescence, such as working memory (WM), because BOLD-activity in the superior parietal cortex shows substantial developmental increases during the manipulation of WM-items [47].

A second region of pronounced changes in IGl-values was the frontal cortex which has been consistently linked to changes in anatomy and behavior during adolescence. In the present study, decreased lGI-values were found in the frontal pole (BA 10), orbitofrontal cortex (BA 11) and the inferior frontal gyrus (BA 47). A large body of work has indicated that these regions are centrally involved in the behavioral modifications during adolescence, such as the improvements in cognitive inhibition [48], risk-taking [49] and mentalizing [50].

Finally, substantial reductions in gyrification were found in a cluster corresponding to BA 19, 20 and 37 which comprises early visual areas and cortical regions dedicated to object recognition. In addition to modifications in higher cognitive functions, adolescence is also associated with improvements in neural oscillations elicited by simple and complex visual stimuli [51], [52] as well as with maturation of object processing in the ventral stream [53].

Strong quadratic effects of age on lGI were found in left superior-frontal (BA 8, 9 and 10) and righthemispheric frontal (BA 8) clusters, which is in line with a previous study by (Hogstrom et al. [22]. Cubic age-lGI relationships are localized in left superior-frontal (BA 8, 9, 10, 11, 45, 46 and 47), superior-parietal (BA 1, 2, 3, 4, 5, 6, 7, 8 and 9), right caudal-middlefrontal (BA 8 and 9) and middle-temporal (BA 21) areas.

The current data thus provide a novel perspective on regions involved in gyrification development during adolescence which overall are characterized by a linear developmental trajectory with some regions showing curvilinear and cubic effects. Previous studies with smaller sample sizes [20], [21] identified predominantly changes in GI-values in temporal, parietal and frontal regions. In addition, Mutlu and colleagues [20] observed a steeper lGI decrease with age in males than females in prefrontal regions which was not confirmed by the present study.

### Development of Cortical Folding during Adolescence: Relationship with GM/WM-change

Several mechanisms have been proposed for the changes in gyrification during development [54]. Van Essen [55] suggested that the folding pattern of the cerebral cortex can be explained by the mechanical tension along axons. According to this theory, the formation of gyri is the result of mechanical forces between densely linked regions as tension pulls strongly interconnected regions together. In addition, alternative accounts emphasized the role of differential growth between inner and outer cortical layers [17]. Finally, there is evidence that cortical folding is under genetic control [56] and that sex-differences exist in the mature cortex [57].

While the current study does not allow insights into the mechanisms underlying the reductions in gyrification during adolescence, comparison with changes in GM- and WM-parameters may be important for the question whether the observed changes in cortical folding are influenced by ongoing anatomical modifications. An important finding of the current study is that the reductions in lGI-values occur in cortical regions which are largely distinct from reductions in the volume and thickness of GM. Correlations between lGI-values in regions which were characterized by pronounced age-dependent decreases and GM/WM-parameters suggest, however, that the degree of cortical folding is nonetheless related to GM-volume and surface area. Specifically, we observed a positive relationship between increased lGI-values with surface area and volume of GM. Interestingly, this was not the case for the thickness of GM. Finally, WM-volume also contributed to higher lGI-values in 5 out of 7 cortical regions.

### Gyrification, Behavior and Psychopathology

Despite the widespread reductions in cortical folding during adolescence and the large effect sizes associated with decreased lGI-values, the implications for changes in cognition and behaviour during adolescence remain to be established. Previous research has indicated that individual differences in cortical folding in frontal regions influence executive processes in adults [58] and behavioral modifications, such as meditation [59], impact on gyrification, suggesting a role of cortical folding in cognition and experience-dependent plasticity.

Furthermore, there is a large body of evidence that gyrification patterns are associated with psychopathology which underlines the potential importance of understanding developmental changes in gyrification and the relationship to cognition and behavior. Several neurodevelopmental disorders, such as Williams Syndrome (WS) and Autism Spectrum Disorders (ASDs), are associated with abnormal cortical folding patterns. Specifically, participants with WS are characterized by reductions in the depth of sulci in parieto-occipital regions which are prominently involved in the visuo-constructive deficits [60]. In contrast, gyrification patterns in ASDs are characterized by increased folding relative to normally developing children [61].

Schizophrenia is a severe psychiatric disorder with a typical onset during the transition from adolescence to adulthood which also involves aberrant gyrification. Post-mortem [62] and MRI-studies [63], [64] observed an increase in cortical folding, especially in the prefrontal cortex, which furthermore is predictive for the development of schizophrenia in at-risk subjects [65]. More recently, folding defects have also been shown to predict poor treatment response in first-episode psychosis [66].

Because our data strongly suggest that cortical folding undergoes major modifications during adolescence, one possibility is that in addition to early neurodevelopmental influences, abnormal brain development during adolescence contributes to the aberrant anatomy of the neocortex and the manifestation of cognitive dysfunctions and clinical symptoms.

## Conclusion

The findings support the view that the adolescence involves fundamental changes in the architecture of the cerebral cortex. Specifically, we can show that cortical folding patterns undergo pronounced change which involves a reduction in gyrification across large areas of the cerebral cortex, in particular in precentral, frontal and temporal regions. Future studies need to establish the functional relevance of these modification for concurrent changes in behavior, cognition and physiology through correlations with neuropsychological data and functional brain imaging methods, such as fMRI and MEG.

## Supporting Information

Figure S1 **Nonlinear age effects on the local gyrification index (lGI) in a whole brain, vertex-by-vertex analyses projected onto an average template brain.** Top Row: Age^2^ effects are illustrated for the left hemisphere (left) and right hemisphere (right) from lateral and medial views. Bottom Row: Correlations between age^3^ and lGI are shown for the left (left) and right hemisphere (right) from lateral and medial views. Blue colors indicate a significant decrease of lGI-values with increasing age, whereas warmer colors are coded for an increase in lGI. All analyses were performed by controlling for the effects of gender, eTIV and age (linear). Note: No significant correlations between age^3^ and lGI were found by controlling for the effects of gender, eTIV, age (linear) and age^2^.
(TIFF)

Figure S2 **Nonlinear age effects on GMV in a whole brain, vertex-by-vertex analyses projected onto an average template brain.** Left: Age^2^ effects on GMV for the left hemisphere from lateral and medial view. Right: Effects of age^3^ are illustrated for the left hemisphere from lateral and medial view. Blue colors indicate a significant decrease of GMV with increasing age, whereas warmer colors are coded for an increase in GMV. All analyses were performed by controlling for the effects of gender, eTIV and age (linear). Note: No significant correlations between age^3^ and GMV were found by controlling for the effects of gender, eTIV, age (linear) and age^2^.
(TIFF)
