# Supplementary figures and images for: Adolescent Brain Maturation and Cortical Folding: Evidence for Reductions in Gyrification
Source: PLoS One. 2014 Jan 15;9(1):e84914. doi: 10.1371/journal.pone.0084914 (PMC3893168; doi:10.1371/journal.pone.0084914)

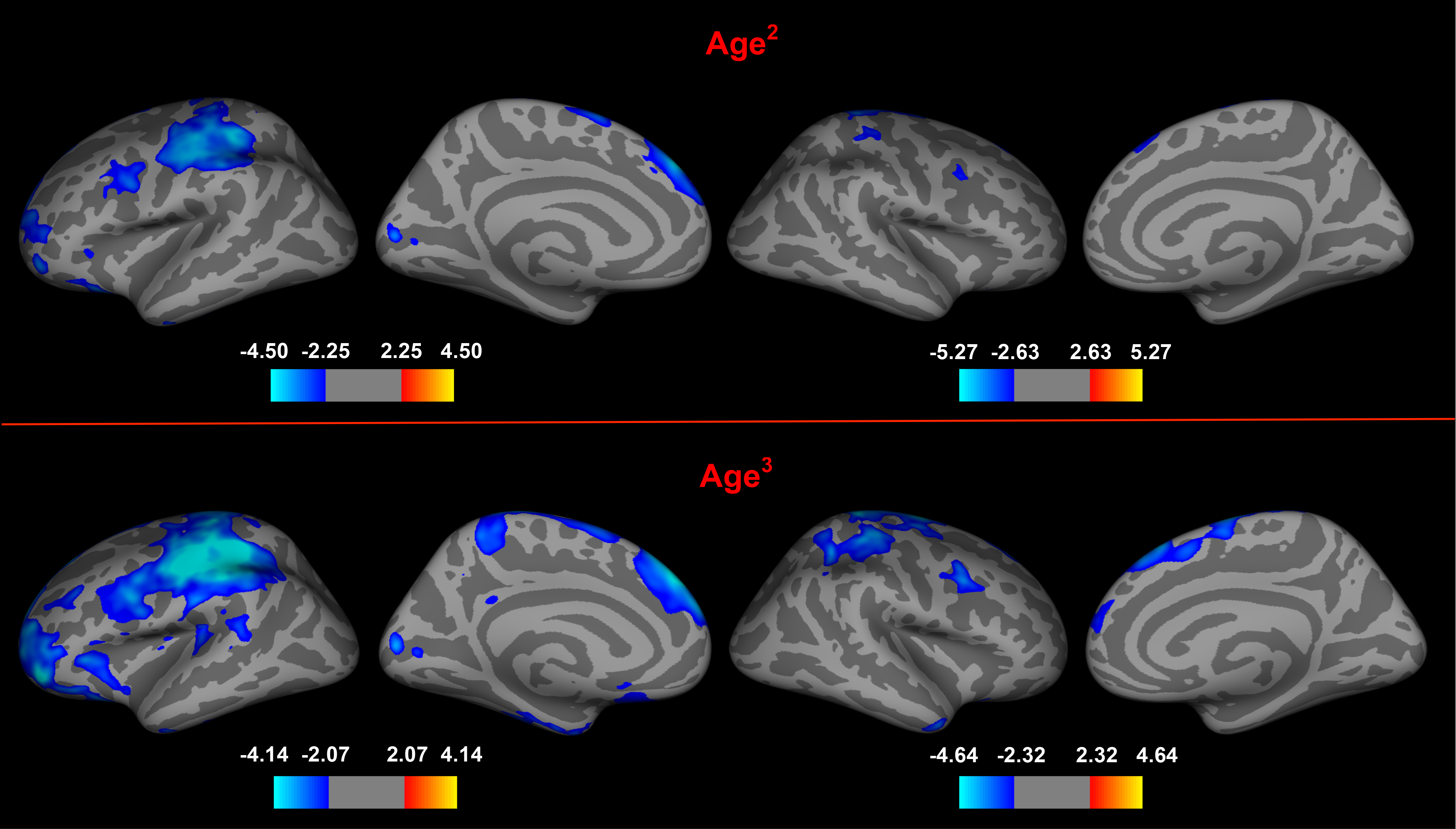

Supplement: Figure S1 — Nonlinear age effects on the local gyrification index (lGI) in a whole brain, vertex-by-vertex analyses projected onto an average template brain. Top Row: Age2 effects are illustrated for the left hemisphere (left) and right hemisphere (right) from lateral and medial views. Bottom Row: Correlations between age3 and lGI are shown for the left (left) and right hemisphere (right) from lateral and medial views. Blue colors indicate a significant decrease of lGI-values with increasing age, whereas warmer colors are coded for an increase in lGI. All analyses were performed by controlling for the effects of gender, eTIV and age (linear). Note: No significant correlations between age3 and lGI were found by controlling for the effects of gender, eTIV, age (linear) and age2. (TIFF) [file pone.0084914.s001.tiff]

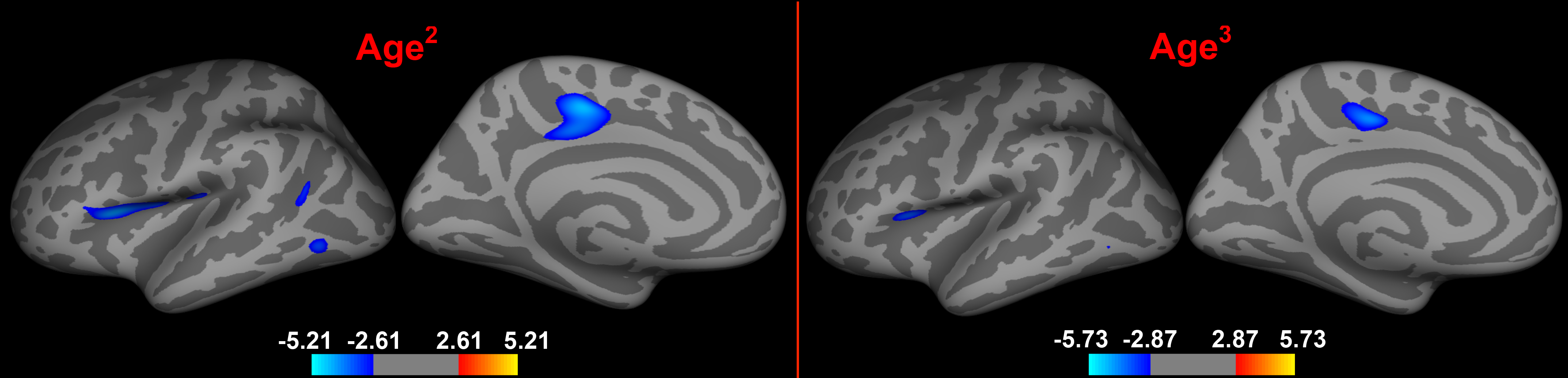

Supplement: Figure S2 — Nonlinear age effects on GMV in a whole brain, vertex-by-vertex analyses projected onto an average template brain. Left: Age2 effects on GMV for the left hemisphere from lateral and medial view. Right: Effects of age3 are illustrated for the left hemisphere from lateral and medial view. Blue colors indicate a significant decrease of GMV with increasing age, whereas warmer colors are coded for an increase in GMV. All analyses were performed by controlling for the effects of gender, eTIV and age (linear). Note: No significant correlations between age3 and GMV were found by controlling for the effects of gender, eTIV, age (linear) and age2. (TIFF) [file pone.0084914.s002.tiff]
